# Influence of adrenergic drugs and volume loading on mean systemic filling pressure in non-septic and septic sheep

**DOI:** 10.3389/fmed.2026.1871295

**Published:** 2026-07-23

**Authors:** Robert G. Hahn

**Affiliations:** Department of Clinical Sciences at Danderyd Hospital, Karolinska Institutet, Stockholm, Sweden

**Keywords:** adrenergic receptors, fluid therapy, norepinephrine, phenylephrine, sepsis

## Abstract

**Objectives:**

To outline the single and combined effects of adrenergic drugs and volume loading on mean systemic filling pressure (P_*msa*_) in septic and non-septic sheep.

**Methods:**

P_*msa*_ was calculated based on cardiac output and the arterial and central venous pressures on 22 occasions during each of 44 experiments in non-septic and septic sheep. A bolus intravenous infusion of 20–25 mL/kg of crystalloid fluid was given together with continuous administration of phenylephrine, norepinephrine, isoprenaline, or dopamine during 150 min. Six non-septic and 5 septic sheep served as controls.

**Results:**

P_*msa*_ attained similar baseline values in the non-septic and septic sheep when no vasopressor was given (17.1 vs. 16.8 mmHg). The non-septic groups that received vasopressor showed higher P_*msa*_ while the baseline values were generally lower in the septic sheep. Fluid loading effectively increased P_*msa*_ in all groups except in the dopamine group with sepsis. Phenylephrine combined with fluid most effectively achieved and maintained a high P_*msa*_ during sepsis. However, the average P_*msa*_ during the 2.5-h experiment was 23% lower in the septic sheep (*P* = 0.007); this difference was mainly due to gradually decreasing P_*msa*_ after volume loading in the noradrenaline group and to low P_*msa*_ in dopamine-treated animals.

**Conclusion:**

P_*msa*_ was lower during early sepsis than in the non-septic state but remained reasonably well preserved after fluid had been infused. The vasopressors exerted only a modest effect on P_*msa*_ during sepsis.

## 1 Introduction

Sepsis is a life-threatening dysregulation of the immune response to infection. Circulatory derangements often require both fluid boluses and adrenergic drugs to maintain cardiovascular performance and organ function (1–4). Adrenergic drugs have clear effects on hemodynamics while the distribution of fluid might be less impacted (5). However, the sub-atmospheric pressure in the interstitium is deepened during sepsis, which creates a “suction pressure” that may negatively impact the intravascular volume status (6).

The ultimate measure of filling of the cardiovascular system is the “mean systemic filling pressure” (P_*ms*_) which represents the pressure that develops in the systemic circulation if the heart suddenly stops (7, 8). In the intact circulation, P_*ms*_ indicates the relationship between the “stressed blood volume,” which is the fraction of the blood volume that exerts pressure on the vascular walls, and the distensibility of the vascular tree. Adequate management of P_*ms*_ is crucial when treating septic patients.

P_*ms*_ is the key parameter in the hemodynamic theory that was created by the physiologist Arthur Guyton in the 1950s. It is built on the view that that the heart fills passively (9, 10). Cardiac output (CO) is then determined by the venous return which, in turn, is driven by the difference between P_*ms*_ and the central venous pressure (CVP).

Algorithms have been developed to predict P_*ms*_ in humans. One analog (P_*msa*_) developed by Parkin and Leaning (11) is based on CVP, CO, mean arterial pressure (MAP), and a reference measurement called *c* that is an anthropometric equation (11, 12). A value of *c* for sheep has been published (13).

The present study explores how P_*msa*_ is affected by adrenergic drugs and if volume loading modifies this relationship. The data used for the analysis was taken from a study in healthy sheep and a similar one performed another is septic sheep (5). The hypothesis was that sepsis and adrenergic therapy alter the influence of intravenous volume loading on P_*msa*_. Parameters closely associated with P_*msa*_ in Guyton’s hemodynamic theory are also reported.

## 2 Materials and methods

### 2.1 Study design

This study is a secondary analysis of two series of experiments; one performed in non-septic and the other in acutely septic sheep. In both studies, the purpose was to monitor the influence of adrenergic drugs on the distribution and elimination of infused crystalloid fluid. The data have previously been used in a kinetic study aimed to demonstrate “third-spacing” of fluid in sepsis (5).

The non-septic study consisted of 24 experiments performed on different occasions, separated by at least 2 days, in 6 awake adult healthy sheep weighing 29–35 kg (mean 32 kg). Each of them underwent four experiments where 24 mL/kg of 0.9% saline was infused over 20 min. An adrenergic drug was given, in random order, by a constant rate intravenous (i.v.) infusion during three of the experiments while no drug was given during the fourth experiment. The drug infusions were initiated 30 min before the fluid bolus was given to allow physiological adjustment to the drug (14).

The sepsis study included of 20 healthy adult sheep weighing 14–26 kg (mean, 20 kg). Due to ethical concerns and possible carry-over effects, each animal underwent only one experiment and were studied under general anesthesia. The animals were intubated via tracheotomy and ventilated at a tidal volume of 10 mL/kg and respiratory rate of 15 breaths per minute. The anesthesia was maintained with 1.5%–2.5% sevoflurane and intermittent doses of sufentanil and cisatracurium.

Sepsis was created by cecal ligation and puncture, combined with a 10-min intravenous infusion of lipopolysaccharide (0.5 mg/kg). The animals were then randomly allocated to receive no drug or one of 3 adrenergic drugs at a constant rate i.v. infusion over 2.5 h, beginning 50 min after the sepsis induction. After an additional 10 min, an i.v. infusion of Ringer’s lactate (20 mL/kg) over 30 min was initiated.

Hence, the non-septic sheep received all drugs on different occasions to limit the biological variability, while each septic sheep only received one drug due to ethical concerns and the risk of carry-over effects. Administration of the adrenergic drugs were initiated before the measurements started to allow steady state to be attained at time zero. They were continued throughout the study, which was ended at 150 min. By contrast, the volume load was administered only during the first 20–30 min of the study to allow examination of the influence of the adrenergic drug on P_*msa*_ at baseline, during infusion, and after infusion.

### 2.2 Adrenergic drugs

In both series, continuous intravenous infusion of 3 μg/kg/min of phenylephrine (Phe) and 50 μg/kg/min of dopamine (Dop) was administered. Moreover, one group in the non-septic sheep study was given 0.1 μg/kg/min isoprenaline (Iso) and, in the septic sheep, 1 μg/kg/min of noradrenaline (NA). The infusions were maintained throughout the experiments.

### 2.3 Measurements

The sheep were fasting from 24 h before the experiments. The non-septic sheep used a femoral catheter to measure MAP, a pulmonary artery catheter to measure CO, and a central venous to measure the CVP. The data were displayed on a Hewlett-Packard Monitor model 78901 A (Andover, MA, USA).

The same hemodynamic variables were measured via the femoral artery and a central venous catheter in the septic sheep, using the FloTrac/Vigileo system (Edwards Lifesciences, Irvine, CA). In both studies, the urine output was quantified via a catheter placed in the bladder.

### 2.4 Guyton’s hemodynamic parameters

The mean systemic filling pressure (P_*msa*_) was derived from measurements of CVP, MAP, and CO, assuming a constant veno-arterial compliance of 24:1 (11, 12):


P=msaaCVP+bMAP+cCO

where *a* = 0.96, *b* = 0.04 (*a* + *b* = 1). The value of c is an anthropometric measure in humans, but Yastrebov et al. found that *c* averaged 2.65 in 7 sheep having an average body weight of 49.4 kg (13). Correcting for the body weights of the present animals yielded *c*-values of 1.71 and 1.06, respectively. P_*msa*_ was calculated in the same way, and on the same occasion, for all studied animals.

The other parameters in Guyton’s hemodynamics are the pressure gradient for venous return (dVR), which is the difference between P_*msa*_ and CVP, the global pumping efficiency (Eh), which is obtained as the ratio of dVR to P_*msa*_ and, finally, the resistance to venous return (RVR), which is calculated as the ratio of dVR to CO.

### 2.5 Statistics

Data are presented as the mean ± SD and differences in hemodynamic parameters between groups evaluated by one-way analysis of variance (ANOVA) followed by the Bonferroni *post hoc* test for subgroup comparisons, using IBM SPSS 30.0.0 for Mac. No data with skewed distribution are reported. *P* < 0.05 was considered statistically significant.

## 3 Results

### 3.1 Mean systemic filling pressure (P_*msa*_)

The baseline P_*msa*_ was almost identical in the two groups when no vasopressor was given, amounting to 17.1 ± 5.9 mmHg in the non-septic sheep and 16.8 ± 7.1 mmHg in the septic sheep. When vasopressors were given, the baseline P_*msa*_ was higher than in the controls in the non-septic sheep. By contrast, P_*msa*_ was usually lower than in the controls when vasopressors were administered to septic sheep (Table 1).

**TABLE 1:** Mean circulatory filling pressure (P_*msa*_) at baseline when vasopressors were given but no fluid had yet been administered.

Study group	No sepsis	Sepsis	*P*-value
Pmsa (mmHg)			
Control	17.1 ± 5.9	16.8 ± 7.1	0.96
**Phe**	19.8 ± 4.6	13.3 ± 4.6	0.043
**Iso**	18.5 ± 1.9	–	–
**NA**	–	16.7 ± 5.2	–
**Dop**	24.5 ± 4.9	15.2 ± 6.2	0.021
dVR (mmHg)			
Control	11.1 ± 1.6	11.8 ± 7.8	0.81
**Phe**	12.0 ± 1.1	13.3 ± 4.6	0.52
**Iso**	15.2 ± 1.5	–	–
**NA**	–	11.3 ± 46	–
**Dop**	16.7 ± 2.8	12.4 ± 6.7	0.18
Eh (no unit)			
Control	0.68 ± 0.13	0.68 ± 0.20	0.96
**Phe**	0.62 ± 0.11	0.58 ± 0.19	0.66
**Iso**	0.82 ± 0.10	–	
**NA**	–	0.81 ± 0.22	
**Dop**	0.69 ± 0.08	0.68 ± 0.13	0.25
RVR (mmHg min/L)			
Control	2.46 ± 0.16	1.30 ± 0.08	0.001
**Phe**	3.06 ± 0.21	1.51 ± 0.23	0.001
**Iso**	2.13 ± 0.05	–	0.001
**NA**	–	1.35 ± 0.14	
**Dop**	2.22 ± 0.26	1.44 ± 0.22	
Urine output (mL)			
Control	834 ± 174	35 ± 38	0.004
**Phe**	1245 ± 292	50 ± 31	0.004
**Iso**	161 ± 75		
**NA**	–	52 ± 48	
**Dop**	1034 ± 464	31 ± 28	0.001

Statistical testing with one-way ANOVA except urine output examined by the Mann-Whitney’s test. The urine output is also shown. Data are the mean ± SD.

**FIGURE 1:**
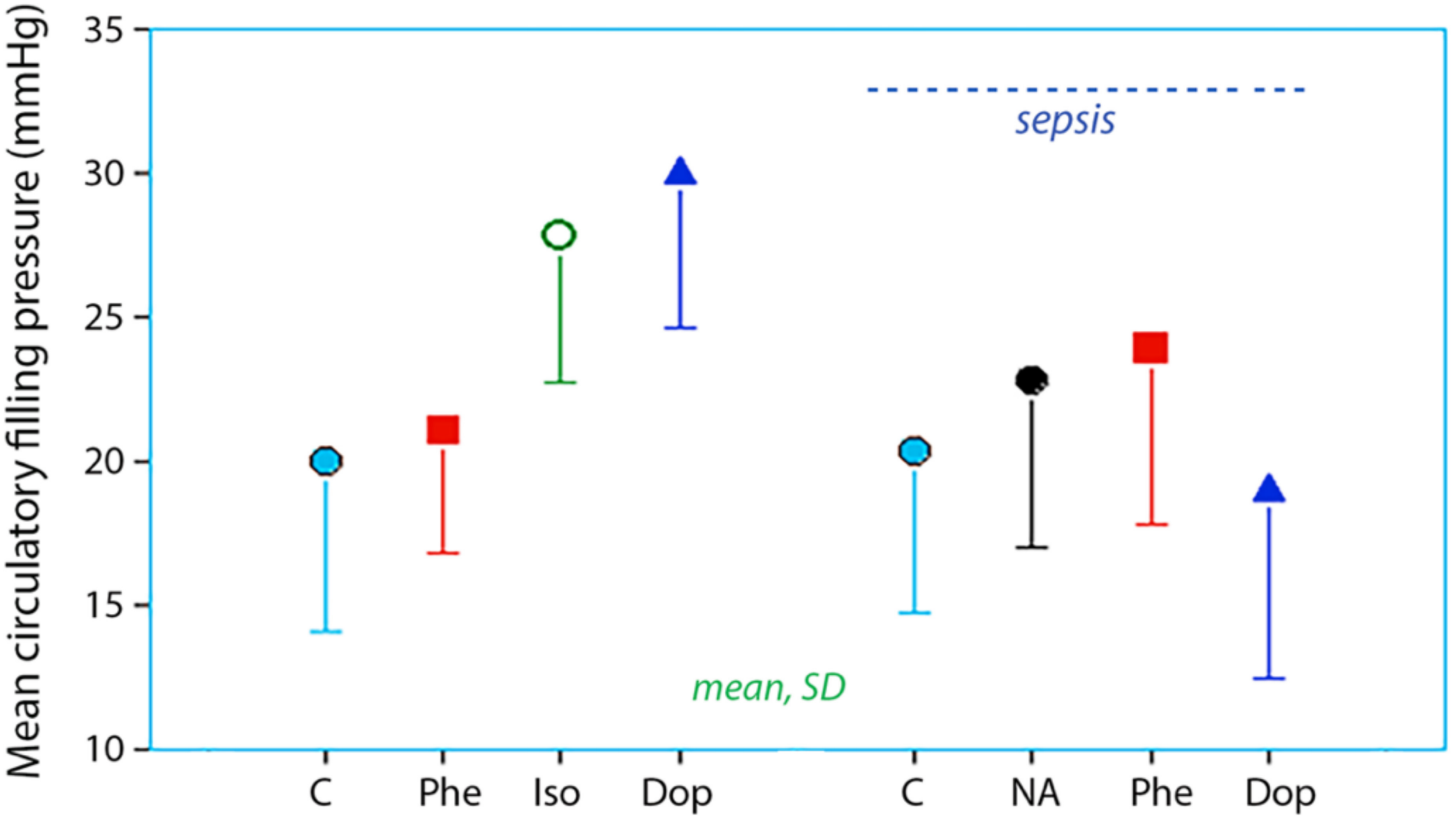
The mean circulatory filling pressure (P_*msa*_) during experiments where various vasopressors were continuously infused during volume loading with crystalloid fluid in non-septic and septic sheep. C, control (no vasopressor); Phe, phenylephrine; Iso, isoprenaline; Dop, dopamine; NA, noradrenaline.

Figure 1 shows the P_*msa*_ for all observations during the experiments. P_*msa*_ was higher with Iso and Dop in the non-septic sheep (*P* < 0.001) while Phe showed no difference compared to the controls. In the septic sheep, Phe induced a higher P_*msa*_ than Dop (*P* < 0.01).

**FIGURE 2:**
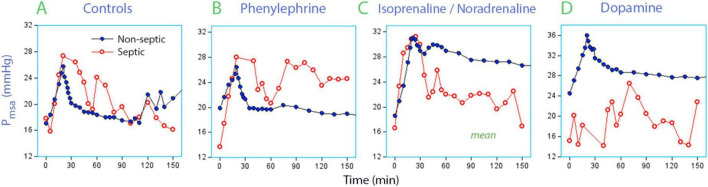
**(A–D)** The trends for mean circulatory filling pressure (P_*msa*_) during experiments where various vasopressors were continuously infused during volume loading with crystalloid fluid in non-septic and septic sheep. The drugs in the C subplot are not fully comparable as the non-septic sheep received isoprenaline and the septic sheep noradrenaline. Mean values.

Figure 2 illustrates trend for the P_*msa*_ during the experiments. The P_*msa*_-time curves were more stable over time in the non-septic shapes and could reach different plateaus before, during, and after the volume load. P_*msa*_ did not differ significantly between the two series of control experiments (Figure 2A) but Phe showed higher P_*msa*_ during sepsis (*P* = 0.031, Figure 2B).

Isoprenaline induced higher P_*msa*_ in the non-septic sheep than NA did in the septic sheep (unmatched comparison, *P* < 0.001; Figure 2C) while Dop yielded markedly lower P_*msa*_ in the septic than in the non-septic sheep (*P* < 0.001; Figure 2D).

### 3.2 Guyton parameters

**FIGURE 3:**
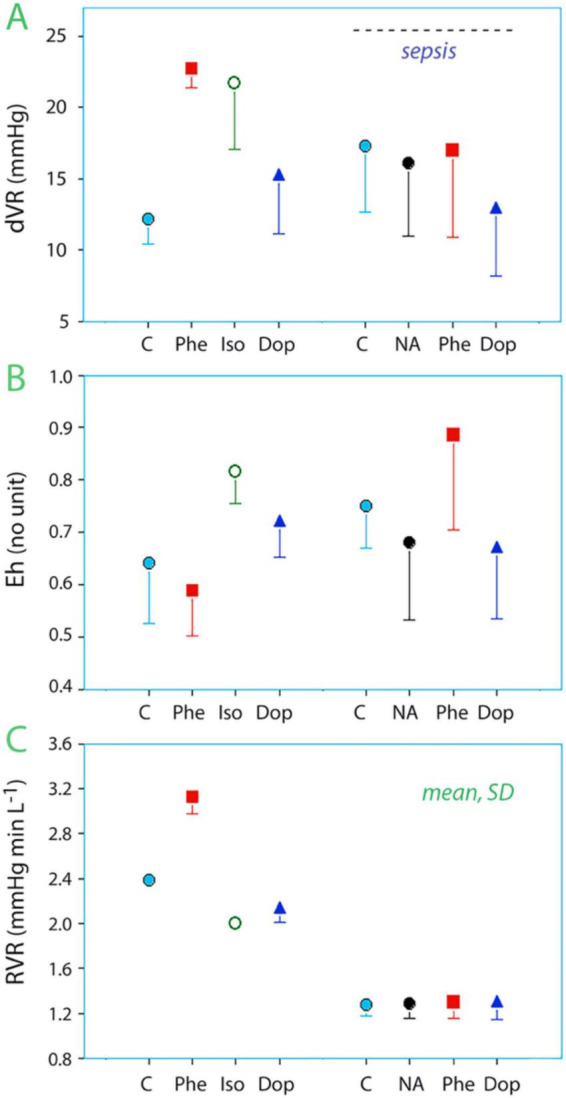
The parameters that complement Pmsa in Guyton’s hemodynamic theory studied in non-septic and septic sheep. **(A)** dVR, pressure gradient for venous return; **(B)** Eh, global pumping efficiency; **(C)** RVR, resistance to venous return. The administered vasopressors were C, control (no vasopressor); Phe, phenylephrine; Iso, isoprenaline; Dop, dopamine; NA, noradrenaline.

The resistance to venous return (dVR) was high in the non-septic Phe and Iso groups (*P* < 0.001 versus the controls) while this was not the case in the septic sheep (Figure 3A).

The global pumping efficiency (Eh) showed a varied picture that did not differ between the septic and non-septic groups (*P* = 0.74) although Phe did yield higher values in the septic state (*P* < 0.001; Figure 3B).

The resistance to venous return (RVR) was very low in the septic sheep regardless of vasopressor (*P* < 0.001 compared to the non-septic sheep; Figure 3C).

Overall, sepsis was associated with lower P_*msa*_ (−23%), dVR (−23%), and RVR (−63%) while the lower Eh in the septic sheep did not reach statistical significance (−3%; Table 2, top).

**TABLE 2:** Guyton’s hemodynamic parameters depending on the type of adrenergic receptor stimulation.

Subgroup	P_*msa*_ (mmHg)	dVR (mmHg)	Eh (no unit)	RVR (mmHg min/L)	CO (L/min)	MAP (mmHg)	CVP (mmHg)
**No sepsis**	20.0 ± 5.1	13.8 ± 2.9	0.71 ± 0.12	2.47 ± 0.41	7.7 ± 3.7	109 ± 28	7.6 ± 3.6
**Sepsis**	15.4 ± 6.4	10.6 ± 5.3	0.69 ± 0.19	1.41 ± 0.19	12.9 ± 4.5	74 ± 17	5.3 ± 3.8
***P*-value**	0.007	0.02	0.70	0.001	0.001	0.001	0.001
No sepsis							
(1) **Control**	20.1 ± 6.9	12.2 ± 1.8	0.64 ± 0.11	2.38 ± 0.12	5.2 ± 1.0	92 ± 7	7.8 ± 4.5
(2) **Alpha-1**	21.1 ± 4.3	12.1 ± 1.3	0.59 ± 0.09	3.13 ± 0.15	3.9 ± 0.5	145 ± 15	9.0 ± 3.6
(3) **Beta-1**	27.8 ± 5.1	22.7 ± 4.6	0.82 ± 0.06	2.00 ± 0.07	11.4 ± 2.6	83 ± 7	5.1 ± 1.9
(4) **Dopamine**	30.0 ± 5.4	21.7 ± 4.1	0.72 ± 0.07	2.14 ± 0.13	10.2 ± 2.3	113 ± 20	8.4 ± 2.7
***P*-value**	0.001	0.001	0.001	0.001	0.001	0.001	0.001
**Subgroup differences**	1, 2 < 3, 4	1, 2 < 3, 4	1, 2 < 3, 4	2 > 1, 3, 4	1, 2 < 3, 4	1, 3 < 2, 4	1, 3 < 2, 4
Sepsis							
(1) **Control**	20.6 ± 5.9	15.3 ± 4.8	.75 ± .16	1.27 ± 0.07	12.1 ± 4.2	72 ± 19	4.7 ± 3.6
(2) **Alpha-1**	23.3 ± 4.3	16.8 ± 4.8	0.72 ± 0.12	1.29 ± 0.11	13.3 ± 4.4	74 ± 16	6.7 ± 3.4
(3) **Beta-1**	22.9 ± 5.9	17.3 ± 4.6	0.75 ± 0.08	1.29 ± 0.10	13.2 ± 5.3	74 ± 14	5.5 ± 2.3
(4) **Dopamine**	19.4 ± 6.6	17.0 ± 6.1	0.88 ± 0.18	1.33 ± 0.14	13.7 ± 4.1	76 ± 18	3.4 ± 3.7
***P*-value**	0.001	0.087	0.001	0.032	0.16	0.43	0.001
**Subgroup differences**	4 < 1 < 2,3	–	1, 2, 3 < 4	1 < 4	–	–	4 < 1 < 2,3

Statistical testing with one-way ANOVA and using the Boferroni *post hoc* test for between group differences. Alpha-1-receptors = Phe and NA; Beta-1 receptors = Iso and NA; Dopamine receptors = Dop. Data are the mean ± SD for all values during the experiment.

### 3.3 Adrenergic receptor effects

The choice of adrenergic drug was not fully comparable between the non-septic and septic sheep. To partly overcome this issue, the study groups were re-defined based on the targeted adrenergic receptor. In the non-septic sheep, stimulation of the alpha-1-receptors by Phe was associated with higher RVR, MAP, and CVP but not with higher P_*msa*_ (Table 2, top). By contrast, stimulation of the beta-1-receptors (with Iso) and the dopamine receptors (with Dop) considerably increased P_*msa*_, dVR, Eh, and CO (Table 2, middle). In the septic sheep, the vasoactive drugs induced only minor hemodynamic changes (Table 2, bottom).

## 4 Discussion

### 4.1 P_*msa*_ and sepsis

The septic condition represented a hyperkinetic state, as evidenced by a 70% higher CO. However, the high CO was balanced by low MAP and CVP, giving P_*msa*_ essentially the same baseline value in the non-septic and septic sheep when a vasopressor was not given. This was not expected as the septic sheep were under general anesthesia, which is known to lower the baseline for P_*msa*_ (15). The low urine output and the fact that sepsis reduces the capillary surface area available for filtration might also have contributed to the well maintained P_*msa*_ (16, 17). Administration of vasopressor altered the baseline by promoting higher P_*msa*_ in the non-septic sheep while, surprisingly, P_*msa*_ attained lower values in the septic sheep. RVR was the only “Guyton parameter” that was lower in the septic sheep at that time.

Mean systemic filling pressure was effectively increased by fluid loading in all groups except in the septic Dop animals. Phenylephrine plus fluid was the combination that best maintained P_*msa*_. However, a slow reduction of P_*msa*_ occurred in the septic sheep over time, which suggests that the fullness of the vascular tree gradually decreased despite low urine output. P_*msa*_ also fluctuated quite much, suggesting cardiovascular instability, while the non-septic sheep exhibited only minor short-term variations that generally seemed to follow the hemodilution.

### 4.2 Effects of adrenergic drugs

Increasing the vascular tone by vasopressors is believed to increase P_*msa*_ by transferring blood from the non-stressed to the stressed pool and/or reducing the compliance of the vascular tree for volume expansion. This functional redistribution within the vascular system counteracted the early and consistent reduction of RVR, which indicated a virtual collapse of the resistance to venous return that occurred regardless of vasopressor therapy. The slow reduction of P_*msa*_ over time in the septic sheep can be attributed to either sepsis-induced desensitization of adrenergic receptors or to maldistribution of fluid due to trapping in the “third fluid space” (6). Sepsis affects the hemodynamics faster and more strongly than it changes the fluid distribution (5), but trapping of fluid into the “third fluid space” does occur, which secondarily reduces the lymphatic flow and further promotes hypovolemia (18).

There were differences in the responses to the vasopressors between the non-septic and septic sheep. The most dramatic difference was found in the Dop group. Here, the drug effectively increased P_*msa*_ at baseline and during the volume loading in the non-septic sheep, while only a vague response could be discerned in the septic sheep. Dop is vasodilatory in low doses, while large amounts exert vasoconstrictive and inotrophic effects. Dop also increases the urine output, which is best known for low doses, but the high dose given in the present study did exert a diuretic effect in the non-septic sheep. However, both the vasoconstrictive and diuretic effects of Dop were absent in the presence of sepsis.

A different response was seen for Phe, which showed a marginal P_*msa*_ response to vasopressor and fluid in the non-septic sheep while the response was much stronger during sepsis. This can possibly be attributed to the great difference in urine flow between the two experimental situations; the urine output even exceeded the infused volume the non-septic Phe sheep, while the septic Phe sheep only excreted a negligible volume. Thus, the fluid balance was much more positive in the septic sheep, which apparently maintained P_*msa*_.

The data then suggest that a continuous infusion of a high dose of Phe would normally promote hypovolemia due to excessive urine flow. The associated dehydration of the extravascular space is then likely to reduce the risk of high-pressure (“push”) third-spacing of fluid (6). By contrast, hypovolemia is counteracted in septic sheep by what seems to be a generalized renal insensitivity to adrenergic stimuli. These animals developed low-pressure (“pull”) third-spacing (5) but sufficient volume to compensate P_*msa*_ for the maldistribution of fluid was apparently provided by the reduced diuresis.

Grading the influence of vasopressors on P_*msa*_ in the septic sheep yields that Phe was most effective, NA took an intermediate position, and Dop was least effective. NA is currently the favored vasopressor for treatment of sepsis in humans. NA rapidly increases and stabilizes MAP and is claimed to increase the stressed blood volume (19). Phe is a pure alpha-1-agonist while NA also stimulates beta-1-receptors, which is a perceived benefit as the latter receptors improve the contractility of the heart. In the present study, both Phe and NA effectively increased P_*msa*_ during the volume loading in the septic sheep, but only Phe maintained the elevation post-infusion. A more pronounced time-dependent attenuation of the adrenergic responsiveness to NA than to Phe might possibly explain this difference.

### 4.3 Guyton’s hemodynamics

The research works by Arthur Guyton from the 1950s link circulatory volume with pressure and flow (9). P_*msa*_ and not the heart serves as the driving pressure, whereas the true driving force is the difference between P_*msa*_ and CVP. Hence, CVP reduces venous return, which governs CO. The flow gradient is greater if P_*msa*_ is high and/or CVP is low, which is expressed by the parameter Eh that then is a measure of how effectively a change of the “stressed” blood volume increases the CO. A low P_*msa*_ and a low CVP makes “fluid responsiveness” more likely (12, 20) but the baseline is moved if vascular compliance is changed, such as when general anesthesia in induced (15).

In the clinic, the mean systemic filling pressure has been measured in three different ways. One is based on suppression of venous return by deep ventilatory inspirations (21, 22). The idea behind the second method is to arrest the circulatory flow in one arm by inflating a blood pressure cuff and then obtain P_*ms*_ when the arterial and venous pressures become equal. The third method is to calculate the P_*msa*_ using the approach used in the present study. Comparisons have found the methods to be interchangeable (23, 24).

More sophisticated laboratory methods for assessing P_*ms*_ were applied in a series of studies by Möller et al. (25). The group measured this pressure using inspiratory maneuvers, right atrial balloon occlusion, ECMO, and cardiopulmonary bypass (26). In a porcine model, they found that the indirect method used in the present study (P_*msa*_) accurately estimated both absolute and changing values of P_*ms*_ (27).

### 4.4 Limitations

This analysis represents a retrospective analysis of two studies that were performed in a similar way. Limitations include a small size of the groups (5–6 animals), making statistics somewhat speculative. The choices of adrenergic drugs in the non-septic and septic sheep were not completely identical. Specifically, Iso was used in the non-septic sheep as an example of a pure beta-1-receptor agonist, while this drug was replaced by NA, which is a mixed alpha-1- and beta-1-agonist, in the septic sheep. These vasopressors were compared in Figure 2C, which can be questioned since Iso is devoid of beta-1-stimulating quality. The original study of septic sheep contained a subgroup given esmolol that is not included in the present report due to lack of a suitable control group.

The blood sampling protocol was essentially the same in both studies although the non-septic study lasted 3 h instead of 2.5 h. The present analysis was limited to 2.5 h in both series. Repeated-measures statistics was not used because occasionally missing measurements would then exclude several experiments.

The non-septic sheep were studied in the awake state while the septic animals, due to ethical concerns, were studied under general anesthesia. This difference is likely to have exaggerated the hemodynamic differences between the groups. The awake sheep also received all drugs on different occasions, which reduced the inter-individual variability, but this approach was not possible for the septic sheep due to ethical concerns and expected carry-over effects.

The veno-arterial compliance might not be constant at 24:1 in both non-septic and septic sheep, which was assumed in the present study. The arterial compliance is known to decrease during sepsis while the venous capacitance becomes compromised (28). This might exaggerate the influence of MAP on P_*msa*_ in the septic sheep and result in a lower effective P_*msa*_. However, details about how these alterations influence calculations of Guyton’s hemodynamic parameters are poorly known. As mentioned above, the present equation used for the calculation of P_*msa*_ has been found to be accurate despite several types of highly invasive mechanical interventions in pigs, suggesting wide applicability (26, 27).

## 5 Conclusion

Vasoactive drugs and crystalloid fluid increased P_*msa*_ in healthy sheep while the response was weaker in septic sheep. The influence of sepsis on Guyton’s hemodynamic parameters was much smaller compared to the conventional hemodynamic variables, such as cardiac output and central venous pressure. Phenylephrine was most effective in raising P_*msa*_ due to a long-lasting effect, noradrenaline took an intermediate position, and dopamine was least effective. Noradrenaline is the preferred vasoconstrictor in septic humans due to its combined beta-1 and alpha-1-receptor stimulating effects but, in the present study, its influence on P_*msa*_ was attenuated over time which did not occur with phenylephrine.

## Data Availability

The original contributions presented in this study are included in this article/Supplementary material, further inquiries can be directed to the corresponding author.
